# Supplementary material for: MetaQTL: a package of new computational methods for the meta-analysis of QTL mapping experiments
Source: BMC Bioinformatics. 2007 Feb 8;8:49. doi: 10.1186/1471-2105-8-49 (PMC1808479; doi:10.1186/1471-2105-8-49)
Supplement: Additional File 7 — MetaQTL Package : jar file and tutorial. This Zip archive contains both the MetaQTL JAR file and the files of the tutorial. [file 1471-2105-8-49-S7.zip › org.inra.metaqtl/doc/org/metaqtl/MetaQtlAnalysis.html]

MetaQtlAnalysis


|  |  |  |
| --- | --- | --- |
| **Overview** \| **Package** \| **Class** \| **Use** \| **Tree** \| **Deprecated** \| **Index** \| **Help** | |  |
| **PREV CLASS**   **NEXT CLASS** | **FRAMES**    **NO FRAMES**     **All Classes** |  |
| SUMMARY: NESTED \| FIELD \| CONSTR \| METHOD | DETAIL: FIELD \| CONSTR \| METHOD |  |


---


## org.metaqtl Class MetaQtlAnalysis

```
java.lang.Object
  org.metaqtl.MetaQtlAnalysis
```

---

``` public class MetaQtlAnalysis extends java.lang.Object ```

A QTL meta analysis consists in a clustering of QTL positions over
chromosomes for different group of traits. The a
`MetaQtlAnalysis` object is just a bundle of chromosomes
in each of which the result of the meta analysis is stored.

---

| **Field Summary** | |
| --- | --- |
| `java.lang.String[]` | `chromNames`             The names of the chromosomes. |
| `int` | `nchr`             The number of chromosomes on which the meta-analysis has been performed. |
| `Qtl[][]` | `qtlByChrom`             The matrix of qtl by chromosome |
| `MetaQtlResult[][]` | `resultByChrom`             The matrix of results where the rows are the chromosomes. |


**Constructor Summary**

`MetaQtlAnalysis(int nchr)`
  
          Creates a new instance of a `MetaQtlAnalysis`


| **Method Summary** | |
| --- | --- |
| `MetaQtlModel` | `getBestModel(int criterion)`             This methods returns the best models for each criterion defined to select the optimal number of clusters per chromosome per trait. |
| `int` | `getChromIdx(java.lang.String chrom)` |
| `Qtl[]` | `getMetaQtl(java.lang.String chrom, java.lang.String trait, int k)`             Returns the meta Qtl for the given chromosome indice `cidx`, the given trait name `trait` and the given number of clusters `k`. |
| `MetaQtlResult` | `getResult(int chromIdx, java.lang.String trait)`             Returns the result for the given trait name or null if not found. |
| `MetaQtlResult` | `getResult(java.lang.String chrom)`             Returns the first result for the given chromosome. |
| `MetaQtlResult` | `getResult(java.lang.String chrom, java.lang.String trait)`             Returns the result for the given trait name or null if not found. |
| `MetaQtlResult[]` | `getResults(java.lang.String chromName)` |
| `int` | `getTraitIdx(int chromIdx, java.lang.String trait)` |

**Methods inherited from class java.lang.Object**

`clone, equals, finalize, getClass, hashCode, notify, notifyAll, toString, wait, wait, wait`

**Field Detail**

### nchr

```
public int nchr
```

:   The number of chromosomes on which the meta-analysis
    has been performed.

---


### chromNames

```
public java.lang.String[] chromNames
```

:   The names of the chromosomes.

---


### qtlByChrom

```
public Qtl[][] qtlByChrom
```

:   The matrix of qtl by chromosome

---


### resultByChrom

```
public MetaQtlResult[][] resultByChrom
```

:   The matrix of results where the rows are the chromosomes.


**Constructor Detail**

### MetaQtlAnalysis

```
public MetaQtlAnalysis(int nchr)
```

:   Creates a new instance of a `MetaQtlAnalysis`

    **Parameters:**: `nchr` - the number of chromosomes on which the meta-analysis has been performed.


**Method Detail**

### getResult

```
public MetaQtlResult getResult(java.lang.String chrom,
                               java.lang.String trait)
```

:   Returns the result for the given trait name or null
    if not found.

    :   **Parameters:**: `trait` - the name of the trait for which the result is asked. **Returns:**: the result or null if not found.

---


### getResult

```
public MetaQtlResult getResult(java.lang.String chrom)
```

:   Returns the first result for the given chromosome.

    :   **Returns:**: the result or null if not found.

---


### getResult

```
public MetaQtlResult getResult(int chromIdx,
                               java.lang.String trait)
```

:   Returns the result for the given trait name or null
    if not found.

    :   **Parameters:**: `trait` - the name of the trait for which the result is asked. **Returns:**: the result or null if not found.

---


### getChromIdx

```
public int getChromIdx(java.lang.String chrom)
```

:   **Returns:**

---


### getTraitIdx

```
public int getTraitIdx(int chromIdx,
                       java.lang.String trait)
```

:   **Returns:**

---


### getMetaQtl

```
public Qtl[] getMetaQtl(java.lang.String chrom,
                        java.lang.String trait,
                        int k)
```

:   Returns the meta Qtl for the given chromosome indice
    `cidx`, the given trait name `trait`
    and the given number of clusters `k`.

    :   **Parameters:**: `cidx` -: `trait` -: `k` -

---


### getBestModel

```
public MetaQtlModel getBestModel(int criterion)
```

:   This methods returns the best models for each criterion
    defined to select the optimal number of clusters per
    chromosome per trait.

    :   **Returns:**

---


### getResults

```
public MetaQtlResult[] getResults(java.lang.String chromName)
```


---


|  |  |  |
| --- | --- | --- |
| **Overview** \| **Package** \| **Class** \| **Use** \| **Tree** \| **Deprecated** \| **Index** \| **Help** | |  |
| **PREV CLASS**   **NEXT CLASS** | **FRAMES**    **NO FRAMES**     **All Classes** |  |
| SUMMARY: NESTED \| FIELD \| CONSTR \| METHOD | DETAIL: FIELD \| CONSTR \| METHOD |  |


---
